# Role of Rate of Force Development in Mediating the Relationship Between Task-Specific Fear and Chronic Low Back Pain Among Japanese Caregivers: A Pilot Cross-Sectional Mediation Study

**DOI:** 10.7759/cureus.98512

**Published:** 2025-12-05

**Authors:** Teppei Abiko, Shin Murata, Hayato Shigetoh, Norihisa Matsumoto, Yamaguchi Hideaki, Michie Ohyama, Eiji Sakata, Wayne Hing

**Affiliations:** 1 Department of Physical Therapy, Faculty of Health Sciences, Kyoto Tachibana University, Kyoto, JPN; 2 Faculty of Health Sciences and Medicine, Bond University, Gold Coast, AUS; 3 Faculty of Rehabilitation, Reiwa Health Sciences University, Fukuoka, JPN; 4 Research Consultancy, Kyoto Tachibana University, Nagoya, JPN; 5 Welfare Equipment, NPO (Non-Profit Organization) Fukushiyogunet, Fukuoka, JPN

**Keywords:** chronic low back pain, kinesiophobia, mediation analysis, rate of force development, sit-to-stand

## Abstract

Background

Chronic low back pain (CLBP) is prevalent among caregivers; however, the mechanisms linking psychological factors like fear of movement to pain are unclear. This study investigated whether the rate of force development (RFD), a measure of explosive strength, mediates the relationship between task-specific fear and CLBP intensity in this high-risk population.

Methods

Thirty-two Japanese care workers (13 CLBP, 19 healthy) performed sit-to-stand and stand-to-sit tasks on a force plate after rating their task-specific fear. Vertical ground reaction force was used to calculate early-phase RFD (0-100 ms) during stand-to-sit, the prespecified mediator. CLBP was defined as pain persisting for >3 months. Associations were analyzed using Spearman's correlation and a 1,000-sample bootstrap mediation model.

Results

Early-phase RFD (0-100 ms) during stand-to-sit correlated negatively with both task-specific fear (ρ = -0.43) and CLBP intensity (ρ = -0.51). The mediation analysis confirmed a significant indirect effect of fear on CLBP through RFD (B = 0.39, 95% CI (0.06, 1.30)). The total effect of fear on pain was also significant (B = 5.58, 95% CI (0.39, 18.61)).

Conclusion

In caregivers, higher task-specific fear is associated with suppressed RFD during the stand-to-sit task, reflecting a force-control deficit linked to CLBP. Early-phase RFD is a promising biomarker for identifying fear-driven motor inhibition. These findings suggest that workplace interventions that combine graded exposure with explosive-strength training may effectively reduce the risk of chronic pain.

## Introduction

Chronic low back pain (CLBP) is a major contributor to occupational disability and economic loss worldwide [1]. It is especially prevalent among individuals in physically demanding occupations, such as healthcare, caregiving [2], manufacturing, and construction [3], where frequent lifting and repetitive trunk movements are common. In these settings, CLBP contributes to absenteeism and job turnover, leading to staff shortages and reduced efficiency. While physical loading is a key factor, psychological contributors, such as kinesiophobia, defined as the fear of movement due to anticipated pain or re-injury, have also been implicated in the development of disabilities [4], a contributing factor of CLBP and work absence [5,6].

Kinesiophobia has been linked to altered lumbar kinematics during movement tasks [7,8]. Individuals with a higher fear of movement often demonstrate slower movement initiation, reduced trunk range of motion, and increased stiffness, particularly during bending or transitional movements [7,9-12]. These behaviors are thought to reflect avoidance strategies aimed at minimizing pain or avoiding perceived risk of injury [13]. In particular, a systematic review reported that slower lumbar movements are observed in CLBP patients [14]. However, such adaptations may lead to inefficient or rigid motor behavior, increased muscular co-contraction, and abnormal loading on spinal structures. Over time, these biomechanical alterations may contribute to the persistence or exacerbation of chronic low back pain.

The sit-to-stand task is a fundamental movement in daily life that requires coordinated neuromuscular control and sufficient lower-limb strength. The rate of force development (RFD) during sit-to-stand, particularly within the early phase, reflects the capacity for rapid force production [15] and is considered a key indicator of neuromuscular responsiveness. Previous studies have shown that reduced RFD during sit-to-stand is associated with impaired physical function and increased fall risk, especially among older adults [15,16]. In individuals with elevated kinesiophobia, movement hesitation, such as delayed initiation [12] and increased trunk stiffness, has been observed and may contribute to diminished rapid movement [8]. Therefore, RFD measurements during sit-to-stand may provide a practical and objective means of detecting movement hesitation linked to altered motor control without the need for motion analysis systems, supporting their potential utility in clinical and occupational health settings. Conceptually, task-specific fear may promote movement hesitation and increased trunk stiffness during transitional tasks, which, in turn, reduces early-phase RFD. Reduced RFD may then contribute to altered spinal loading patterns and the persistence of CLBP. This hypothesised pathway, linking task-specific fear to CLBP through reduced early-phase RFD, formed the basis for the mediation model examined in the present study.

Although previous studies have shown that kinesiophobia is associated with both CLBP and altered motor behaviour [17], including slowed motor performance, the potential mediating role of RFD in this relationship has not been directly examined. RFD reflects the ability to rapidly generate muscular force, and its reduction may indicate neuromuscular adaptations linked to fear-related movement patterns such as hesitation and increased stiffness. These adaptations, while intended to avoid pain, may contribute to the persistence of CLBP.

To investigate this mechanism, we utilized mediation analysis to test whether RFD serves as an intermediate variable in the relationship between kinesiophobia and CLBP. Clarifying this pathway could enhance our understanding of how psychological and neuromuscular factors interact in chronic pain. Eccentric control tasks, such as stand-to-sit transitions, where rapid force dissipation is critical, may be especially sensitive to fear-driven motor adaptations. Moreover, if RFD is shown to partly mediate this relationship, it may support the utility of exercise-based interventions aimed at improving motor control as a strategy to reduce kinesiophobia and alleviate chronic pain.

Repeated patient handling exposes caregivers’ lumbar spines to compressive and shear loads [18]. Such loading may blunt the early phase of RFD, amplifying fear-driven motor inhibition and ultimately chronic pain. Clarifying this occupation-specific pathway is critical to support both prevention and rehabilitation in caregiving settings. Given the limited existing evidence and exploratory nature of this research area, we conducted a pilot study to preliminarily examine these mediation pathways among Japanese caregivers.

This study aimed to examine whether the relationship between kinesiophobia and CLBP is mediated by RFD during sit-to-stand and stand-to-sit tasks, in addition to the primary analysis in the CLBP group. We hypothesised that lower early-phase RFD, particularly during the stand-to-sit movement, would mediate the positive association between task-specific fear and CLBP in Japanese female caregivers.

## Materials and methods

Participants 

This study included two groups of female formal caregivers: individuals with CLBP, defined as having low back pain persisting for more than three months, and individuals without any current or prior history of low back pain, defined as normal. Participants were excluded if they had low back pain lasting less than three months; a history of spinal surgery; radicular symptoms or neurological deficits in the lower limbs; a history of vertebral compression fracture, spinal tumour, infection, inflammatory spondyloarthritis, or other specific spinal pathology; diagnosed systemic neurological or musculoskeletal disorders; lower-limb pain affecting functional mobility; cognitive impairment that interfered with task compliance; or an inability to independently perform sit-to-stand and stand-to-sit tasks.

Of the 49 care workers initially recruited, 32 participants were included in the final analysis based on the availability of complete data for the planned mediation models. Thirteen participants had CLBP, and the remaining 19 were normal. All participants provided written informed consent prior to participation. The study protocol was approved by the Kyoto Tachibana University Ethics Committee (Approval No. 23-02).

Sample size

This investigation was planned as an external pilot to inform a future longitudinal trial. Pilot guidelines recommend ≥12 participants per group [19] and, for small, standardized effects (δ ≤ 0.3), ≥15 [20]. Our final sample (CLBP n = 13, pain-free n=19) meets the former and approaches the latter. To gauge statistical sensitivity, we ran a Monte-Carlo power simulation in R (10000 replications; path coefficients βa = βb = -0.45, residual σ² = 1; joint-significance α = .05). The model indicated that approximately 60 achieves 80 % power to detect an indirect effect |ab| = 0.20, whereas n = 39 yields approximately 60% power-acceptable for feasibility aims and sufficient to estimate design parameters for a definitive trial.

Kinesiophobia: task-specific fear numeric rating scale (TSF NRS)

Participants were instructed to perform a standardized lumbar movement task comprising three sequential phases. They initiated forward lumbar flexion from a natural standing posture as quickly as possible in response to an auditory cue, bent forward to the point of maximal lumbar flexion as if attempting to touch the floor with their hands, and then extended the lumbar spine to return to the upright position [21].

Immediately after completing the movement sequence, participants were asked, “How fearful do you feel about this movement?” Task-specific fear (TSF) was assessed using an 11-point numerical rating scale ranging from 0 (no fear) to 10 (extreme fear) [22], evaluating the subjective fear in relation to the specific motor task just performed.

Rate of force development (RFD)

During both the sit-to-stand and stand-to-sit tasks, vertical ground-reaction force (vGRF) was recorded at 100 Hz using a portable force plate (Biosignalsplux, PLUX Wireless Biosignals S.A., Lisbon, Portugal). The chair height was individually adjusted so that, in the initial sitting position, the hip and knee joints were flexed to approximately 90°. Participants crossed their arms over their chests and did not use their upper limbs for support. They were instructed to stand up and sit down as quickly as possible. Foot position was not constrained; participants were allowed to use their preferred stance width. The vGRF signal was smoothed with a five-point moving average to reduce noise. Integrated vGRF values were then used to compute rates of change over 100-ms and 200-ms intervals.

**Figure 1:**
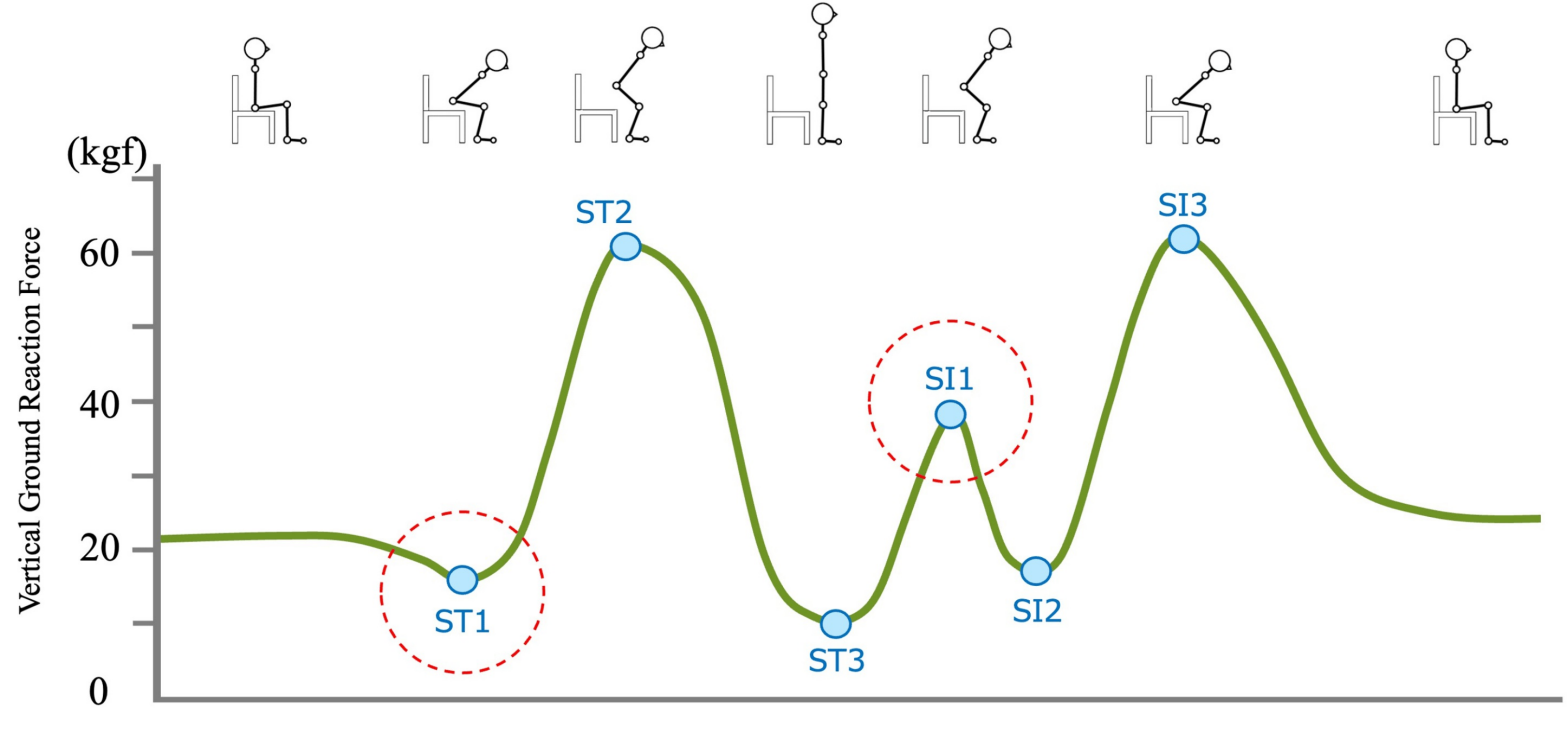
Conceptual diagram of the tested mediation model ST1: sit-to-stand onset, ST2: forward weight-shift peak, ST3: standing-posture onset; SI1: stand-to-sit onset, SI2: pre-seat braking onset, SI3: braking peak before seat contact This figure was created by the authors.

Based on the smoothed signal, integrated vGRF values were used to calculate rates of change over 100-ms and 200-ms intervals. The ratio of these rates was computed at each point to generate a velocity change ratio curve [23]. A local maximum exceeding 1.5 on this curve was defined as point a, and the first subsequent local minimum below 0.85 as point b. The maximum vGRF value between points a and b was identified as the standing timepoint (ST2). The minimum within one second prior to ST2 was defined as ST1, marking the initiation of the rising movement. The first minimum after ST2 was identified as ST3. From ST3, the first subsequent maximum was defined as sitting timepoint1 (SI1), indicating the start of the sitting movement. The next minimum after 1 was defined as SI2. These feature points were used to derive the sit-to-stand and stand-to-sit indicators presented in Figure 1.

RFD was calculated to quantify the rapidity of force generation or reduction during the sit-to-stand and stand-to-sit tasks. It was defined as the change in vGRF over a given time interval, divided by the duration of that interval in seconds and body weight. Specifically, the difference in vGRF between the start and end of each predefined time window was used for this calculation.

In the sit-to-stand movement, RFD was calculated from the initiation point (ST1) over four time intervals: 0-50 ms (RFD_St_0-50), 0-100 ms (RFD_St_0-100), 0-200 ms (RFD_St_0-200), and 100-200 ms (RFD_St_100-200). In the stand-to-sit movement, RFD was similarly calculated from SI1, resulting in RFD_Si_0-50, RFD_Si_0-100, RFD_Si_0-200, and RFD_Si_100-200. In addition to RFD, the peak vGRF during each task (St_Max for standing, Si_Max for sitting) and the time required to reach this peak from the initiation point (St_MaxTime and Si_MaxTime, respectively) were also computed.

Data analysis

Descriptive statistics were calculated for participant characteristics and outcome measures. Associations between TSF NRS and biomechanical outcomes were explored using Spearman’s rank correlation. This exploratory step guided the selection of candidate mediators for subsequent models.

Mediation was tested in two steps. First, a linear regression model estimated the effect of TSF NRS on RFD. Second, a logistic regression model examined whether RFD and TSF NRS predicted CLBP. The indirect effect (a × b), direct effect (c’), and total effect were computed accordingly [24]. Given the small sample size, 1000-sample nonparametric bootstrapping (boot package, ordinary resampling, seed = 1234) generated confidence intervals (CIs). Percentile 95% confidence intervals were used for all path coefficients, including the indirect effect (a × b), direct effect (c′), and total effect.

A pathway was considered significant when its CI excluded zero. P values were omitted, in line with the exploratory nature of this study. Analyses were run in R 4.5.0 with boot, dplyr, readr, tidyr, and tibble packages.

## Results

Participant characteristics and variables are summarized in Table 1. Spearman’s rank correlation analysis revealed a positive association between CLBP and TSF NRS scores (ρ = 0.425, p = 0.015) (Table 2). Regarding RFD during the stand-to-sit task, both RFD_Si_0-50 and RFD_Si_0-100 were negatively correlated with CLBP (ρ = −0.45, p = 0.010; ρ = −0.51, p = 0.003) and TSF NRS (ρ = −0.40, p = 0.025; ρ = −0.43, p = 0.015).

**Table 1:** Participant characteristics and sit-to-stand/stand-to-sit kinetic variables (n = 32) CLBP = Chronic Low Back Pain; LBP = Low Back Pain; TSF NRS = Task-Specific Fear Numerical Rating Scale; RFD_St_xxx = Rate of Force Development during sit-to-stand index; RFD_Si_xxx = Rate of Force Development during stand-to-sit index; kg·s⁻¹·kg⁻¹ = body-weight-normalised force change per second; St_Max = peak vGRF between ST1 (rising onset) and ST2; St_MaxTime = time from ST1 to St_Max (s); Si_Max: peak vGRF between SI1 (sitting onset) and SI2; Si_MaxTime: time from SI1 to Si_Max (s)

Variable	Unit	Average	SD
Years	yo	43.09	13.10
Height	cm	158.16	6.01
Weight	kg	50.64	6.61
CLBP	CLBP/total	13/32	NA
TSF NRS (0-10)	point	0.66	1.63
RFD_St_0-50	kg·s⁻¹·kg⁻¹	0.62	0.65
RFD_St_0-100	kg·s⁻¹·kg⁻¹	2.05	2.18
RFD_St_0-200	kg·s⁻¹·kg⁻¹	4.48	1.94
RFD_St_100-200	kg·s⁻¹·kg⁻¹	6.91	2.63
St_Max	kg/weight	1.48	0.27
St_MaxTime	second	0.26	0.07
RFD_Si_0-50	kg·s⁻¹·kg⁻¹	1.88	1.76
RFD_Si_0-100	kg·s⁻¹·kg⁻¹	2.14	1.57
RFD_Si_0-200	kg·s⁻¹·kg⁻¹	1.68	1.16
RFD_Si_100-200	kg·s⁻¹·kg⁻¹	1.23	1.66
Si_Max	kg/weight	1.03	0.38
Si_MaxTime	second	0.19	0.10

**Table 2:** Spearman correlations among CLBP, TSF NRS, and RFD ρ = Spearman’s rank correlation coefficient; CLBP = Chronic Low Back Pain; TSF NRS = Task-Specific Fear Numerical Rating Scale; RFD_St_xxx = Rate of Force Development during sit-to-stand index; RFD_Si_xxx = Rate of Force Development during stand-to-sit index; St_Max = peak vGRF between ST1 (rising onset) and ST2; St_MaxTime = time from ST1 to St_Max (s); Si_Max: peak vGRF between SI1 (sitting onset) and SI2; Si_MaxTime: time from SI1 to Si_Max (s)

Variable	CLBP	TSF NRS
CLBP	1	0.43*
TSF NRS (0-10)	0.43*	1
RFD_St_0-50	-0.34	-0.22
RFD_St_0-100	-0.35	-0.16
RFD_St_0-200	-0.23	-0.18
RFD_St_100-200	-0.01	-0.14
St_Max	-0.26	-0.34
St_MaxTime	0.23	-0.01
RFD_Si_0-50	-0.45**	-0.40*
RFD_Si_0-100	-0.51**	-0.43*
RFD_Si_0-200	-0.20	-0.30
RFD_Si_100-200	0.13	-0.08
Si_Max	0.18	0.41
Si_MaxTime	0.27	-0.29

Mediation analyses were conducted to examine whether RFD during sit-to-stand and stand-to-sit movements mediated the relationship between TSF NRS and CLBP. In the model with RFD_St_100 as the mediator, the indirect effect (B = 0.66, SE = 10.24, 95% CI (−0.04, 24.50)) was not statistically significant, while the direct (B = 12.93, SE = 14.63, 95% CI (0.10, 24.50)) and total effects (B = 13.59, SE = 15.40, 95% CI (0.30, 25.32)) were significant.

**Figure 2:**
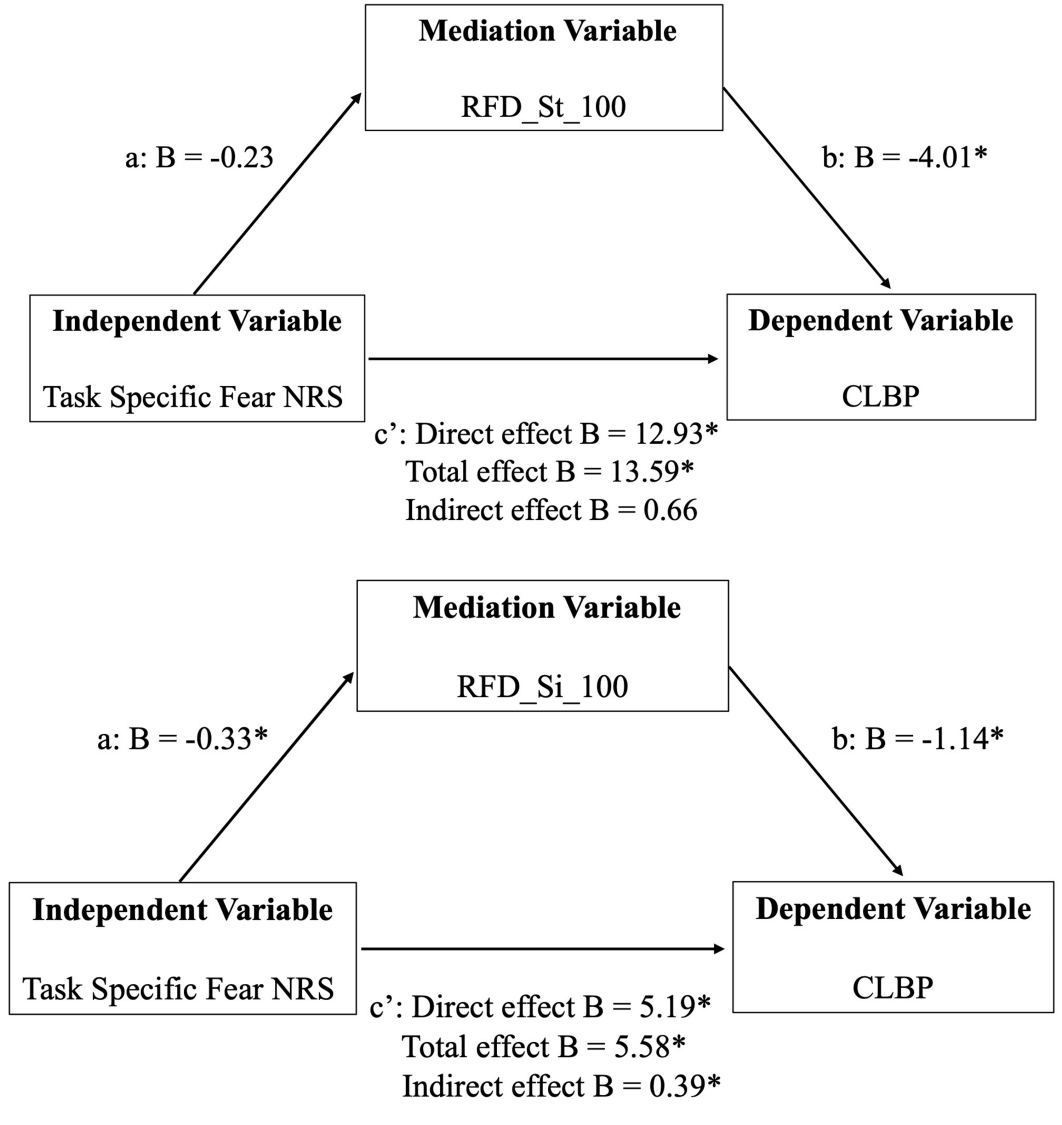
Results of the mediation model Mediation model: Path ‘a’ represents the effect of task-specific fear on RFD; path ‘b’ represents the effect of RFD on CLBP; path ‘c′’ denotes the direct effect of task-specific fear on CLBP, controlling for RFD. Standard errors and confidence intervals for all effects were estimated using 1000 bootstrap samples. Significant paths are indicated by an asterisk.

In the model with RFD_Si_100 as the mediator, the indirect effect (B = 0.39, SE = 0.15, 95% CI (0.06, 1.30)) and the total effect (B = 5.58, SE = 7.06, 95% CI (0.39, 18.61)) were statistically significant, whereas the direct effect was not (B = 5.19, SE = 7.06, 95% CI (−0.17, 18.26)) (Table 3, Figure 2).

**Table 3:** Mediation models linking TSF NRS to CLBP via RFD CLBP = Chronic Low Back Pain; TSF NRS = Task-Specific Fear Numerical Rating Scale; RFD_St_100 = Rate of Force Development within the first 0–100 ms of the sit-to-stand task; RFD_Si_100 = Rate of Force Development within the first 0–100 ms of the stand-to-sit task

		B	SE	95% CI Lower	95% CI Upper
Sit-to-Stand	TSF NRS →RFD_St_100	-0.23	0.18	-0.58	0.05
	RFD_St_100→CLBP	-4.01	70.66	-3.30	-0.06
	Direct	12.93	144.63	0.10	24.50
	Indirect	0.66	10.24	-0.04	0.94
	Total effect	13.59	154.40	0.30	25.32
Stand-to-Sit	TSF NRS →RFD_Si_100	-0.33	0.15	-0.81	-0.15
	RFD_Si_100→CLBP	-1.14	0.91	-2.75	-0.34
	Direct	5.19	7.06	-0.17	18.26
	Indirect	0.39	0.43	0.06	1.30
	Total effect	5.58	7.06	0.39	18.61

## Discussion

Caregivers are repeatedly exposed to patient-handling tasks that require forward trunk bending, making them an ideal population for examining fear-related neuromuscular adaptations in CLBP. This pilot study, therefore, investigated whether TSF is associated with CLBP via neuromuscular inhibition, quantified by RFD. The analysis revealed that reduced RFD during the stand-to-sit task mediated the relationship between TSF NRS and CLBP, whereas no mediation was observed for the sit-to-stand task.

The preliminary correlation analysis indicated that the early phase of RFD measured during the stand-to-sit phase was the only neuromuscular index that correlated with both TSF NRS and CLBP. This finding suggests that higher kinesiophobia may be associated with a reduced early burst of trunk extensor force required for controlled trunk flexion and postural adjustment. In contrast, indices derived from the standing task, absolute peak force, and latency to peak force in both sit-to-stand and stand-to-sit trials showed no meaningful association with fear or pain. Early-phase RFD during the stand-to-sit movement, therefore, appears to be a more sensitive marker of fear-related motor inhibition in CLBP than other metrics measured in this study, such as absolute peak force and latency to peak force.

Mediation analysis indicated that RFD during the stand-to-sit task reflects a motor pattern that partially explains the association between TSF NRS and CLBP. Notably, in the stand-to-sit model, the indirect effect via early-phase RFD accounted for approximately 7% of the total effect. This modest proportion, together with the relatively wide confidence intervals, indicates that the mediating role of RFD should be interpreted cautiously and in the context of the exploratory nature of this pilot study. This finding aligns with prior studies identifying psychological factors as dominant influences in chronic pain, with motor adaptations, such as RFD, contributing to a lesser but measurable extent [25]. The ability to capture this fear-related motor adaptation using a simple kinetic measure highlights the potential utility of RFD as an exploratory biomarker in clinical and occupational settings.

Previous kinematic studies have shown that higher TSF is associated with greater movement hesitation before motion initiation and a longer transition from flexion to extension in individuals with CLBP [12]. Our data add a further implication: once descent began, the first 100 ms of force production (RFD_Si_0-100) was markedly lower in participants with higher fear ratings. This suggests that fear may influence how rapidly the extensor muscles generate force immediately after movement initiation rather than only the overall timing of movement. The distinction between angular-velocity metrics used in earlier work and the RFD index clarifies this difference more precisely [26-28]. Angular velocity captures the speed of a limb already in motion, whereas RFD quantifies the initial force burst that overcomes inertia. Increased trunk stiffness and bilateral co-contraction [9,29], typical protective strategies in fearful individuals, could limit rapid force application without necessarily altering peak force or overall movement time. The stand-to-sit task demands a rapid shift from weight acceptance to controlled descent. Any inhibition within the first 100 ms is therefore functionally significant and, as demonstrated by our mediation analysis, may partially account for the link between TSF and CLBP.

Early force production in the stand-to-sit movement was inversely related to TSF NRS and partly mediated its link with CLBP, whereas the corresponding index for the sit-to-stand movement showed neither association nor mediation effect. Two factors may explain this difference. First, the stand-to-sit movement, which involves forward bending, is integral to high-risk caregiving tasks such as bed-to-chair transfers, dressing, and toileting. This posture places high compressive and shear loads on the lumbar spine [18,30] and was also the specific position used for our fear rating. Therefore, its biomechanical and psychological context aligns with the stand-to-sit phase but not with the upward rise. Second, the sit-to-stand movement rapidly unloads the spine and may be executed through a well-learned extension synergy, leaving less opportunity for fear-related stiffening to suppress the initial force burst. Consequently, only the stand-to-sit task captured the influence of TSF on early force generation and pain.

This study has several limitations. The cross-sectional design does not allow causal inferences, and the observed associations between task-specific fear, early-phase RFD, and CLBP should be interpreted accordingly. The work was conducted as an external pilot with only 13 participants in the CLBP group, resulting in limited power and wide confidence intervals; the mediation findings are therefore preliminary. In addition, BMI, core strength, and detailed postural characteristics were not assessed. Although seat height was individually adjusted and RFD values were normalized to body weight, residual effects of anthropometry and posture cannot be excluded. The sample was restricted to Japanese female caregivers, which may limit generalizability to other populations. Finally, the absence of motion analysis and electromyography prevented a more comprehensive description of trunk kinematics and muscle activation patterns underlying fear-related motor adaptations.

## Conclusions

This pilot study provides preliminary evidence that lower RFD within the first 100 ms of the stand-to-sit movement may partially mediate the association between higher task-specific fear and CLBP. This pattern suggests a complex relationship in which fear-related neuromuscular adaptations may either contribute to the development of chronic pain or arise as a consequence of it. Because RFD can be measured with a single force plate, it may offer an accessible metric for assessing fear-related motor inhibition in occupational settings. Integrating graded exposure with explosive-strength training could therefore be explored as a strategy to improve rapid force control and to identify caregivers who may be at heightened risk of CLBP.
